# The investment case for folic acid fortification in developing countries

**DOI:** 10.1111/nyas.13527

**Published:** 2018-01-24

**Authors:** John Hoddinott

**Affiliations:** ^1^ Division of Nutritional Sciences and Charles H. Dyson School of Applied Economics and Management Cornell University Ithaca New York

**Keywords:** folic acid fortification, neural tube defect prevention, economics, disability‐adjusted life years, cost per death averted

## Abstract

There is compelling evidence that neural tube defects can be prevented through mandatory folic acid fortification. Why, then, is an investment case needed? At the core of the answer to this question is the notion that governments and individuals have limited resources for which there are many competing claims. An investment case compares the costs and benefits of folic acid fortification relative to alternative life‐saving investments and informs estimates of the financing required for implementation. Our best estimate is that the cost per death averted through mandatory folic acid fortification is $957 and the cost per disability‐adjusted life year is $14.90. Both compare favorably to recommended life‐saving interventions, such as the rotavirus vaccine and insecticide‐treated bed nets. Thus, there is a strong economic argument for mandatory folic acid fortification. Further improvements to these estimates will require better data on the costs of implementing fortification and on the costs of improving compliance where regulations are already in place.

## Introduction

Neural tube defects (NTDs) are a significant cause of early‐life (neonatal, infant, and under‐five) mortality.^1^, ^2^ The two most common forms of NTDs, spina bifida and anencephaly, can be prevented by the consumption of folic acid during preconception and early pregnancy.^2^, ^3^, ^4^ Folic acid fortification has proved to be effective in reducing mortality and morbidity associated with NTDs.^5^ Mandatory folic acid fortification is common in many developed countries,^6^ and there exists guidelines and recommendations for doing so.^7^ Why then do developing countries need an investment case for an intervention that saves lives? At the core of the answer to this question is the notion that governments and individuals have limited resources for which there are many competing claims. Consider a government that can invest resources (money and time) in supporting folic acid fortification or purchasing insecticide‐treated bed nets that reduce child mortality due to malaria. Both can save lives, but, given a limited budget, will more lives be saved by investments in folic acid fortification or by buying and distributing bed nets? An investment case attempts to shed light on questions such as these. If folic acid fortification compares well against alternatives, there is a strong investment case for expanding folic acid fortification in developing countries.

## Materials and methods

The investment case for folic acid fortification requires information on the benefits of folic acid fortification, the costs associated with implementing fortification, and a means of bringing the two together in such a way that it is possible to compare these against alternative interventions.

### Benefits of folic acid fortification

There is an extensive literature documenting the impact of folic acid fortification on NTDs; however, there is considerable variation in the magnitude of these impacts across and within countries.^2^, ^5^, ^8^ Furthermore, the vast majority of these come from the United States and Canada, with a smaller number from heavily urbanized countries in Central and South America. In terms of developing the investment case, urbanization matters. Urban consumers are more likely to purchase processed foods, such as wheat or maize flour, which are the primary vehicles for fortification for folic acid fortification. Rural consumers are less likely to consume these products, because they are more likely to consume their own production of staple foods, because, to the extent that they are poorer, they are more likely to consume cheaper coarse grains (such as millet) or other staples that are not processed (e.g., cassava) or because they purchase flours from small‐scale mills that are unable to fortify foods. Sayed *et al*. provide information on the impact of folic acid fortification for South Africa,^9^ a middle‐income country but one that is, relative to the others discussed by Castillo‐Lancelotti *et al*.,^8^ poorer and somewhat less urbanized (at the time of the study, approximately 60% of South Africa's population lived in urban areas). A further attraction in the South African data is that the vast majority of births occur in a health facility where there is a standardized system for reporting NTDs. Sayed *et al*. report that, in South Africa, between the periods January 2003–June 2004 and October 2004–June 2005, the prevalence of spina bifida fell from 0.93/1000 births to 0.54/1000 births, and the prevalence of anencephaly fell from 0.41/1000 births to 0.37/1000 births following the introduction of mandatory folic acid fortification in October 2003 (note that the prefortification period “included an allowance of 9 months for gestation following the introduction of fortification, such that none of these births could have been exposed to fortified foods in the periconceptional period.”) This reduction of 0.43/1000 births is lower than those reported for other countries by Castillo‐Lancelotti *et al*.^8^

A smaller literature focuses on the monetary benefits associated with averted NTDs. These show wide differences across countries. Grosse *et al*. estimate these in terms of the lifetime direct cost savings per live spina bifida case in 2014 dollars for the United States.^10^ These included averted healthcare costs ($513,500); the provision of special education and development services ($63,500); and the opportunity cost of caregiver time ($214,900). These costs total $791,000. They exclude the potential loss in earnings for people living with NTDs compared with a person without and earnings lost due to premature mortality as a result of NTDs. Jentink *et al*. estimate that, in the Netherlands, each NTD child is associated with lifetime costs of €243,000 ($308,000) using a 0% discount rate or €129,000 ($163,000) using a 4% discount rate in 2005.^11^ These costs include health care, special education, and productivity losses but not opportunity costs of caregiver time. Sayed *et al*. report that, in South Africa, the direct medical costs for treating a child with spina bifida in the first 3 years of life were approximately $15,000.^9^

### Costs associated with folic acid fortification

There are two broad categories of considerations: (1) upfront costs associated with the introduction of folic acid fortification and (2) ongoing or recurrent costs. Upfront costs include the purchase of machinery needed to add folic acid, training staff in how to use this machinery, start‐up costs associated with establishing systems for monitoring and compliance, costs associated with social marketing and outreach, and costs arising from the rebranding of these new products and designing labels and containers that meet regulations associated with fortification.^12^, ^13^ There may be costs associated with discarding unfortified products that has already been packaged and labeled and with unused labels. This was an issue in Australia.^12^ However, these costs could be averted if sufficient time is given for stocks to be sold before the introduction of fortification. Ongoing costs include costs (labor and utilities) associated with adding pre‐mix, testing and compliance with food fortification legislation, periodic retraining of staff, ongoing social marketing and outreach activities, and the cost of the folic acid itself. These costs may be borne by the public sector (governments, external donors) and/or by the private sector. Within the private sector, the extent to which these costs are borne by firms (millers, wholesalers, retailers) or households will depend on the extent to which costs associated with fortification are absorbed by firms in terms of reduced profits or whether they are passed onto consumers in the form of higher prices. Note that some of these costs can be shared if fortification with folic acid is done in tandem with fortification with other micronutrients.

While folic acid can be added at different stages of food processing, it is most cost‐effective to do so during the production of flour.^12^ Two studies provide data on upfront and recurrent costs. Access Economics report that the upfront costs for nationwide folic acid fortification in Australia in 2005 were AUS $2,500,000 or $1,875,000. Recurrent costs were estimated to be AUS $1,000,000 for the purchase of folic acid added to all bread‐making flour (used at a concentration of 100 μg folic acid/100 g flour) and AUS $150,000 for public sector costs associated with ensuring compliance—$862,000 in total.^12^ To contextualize these costs, at the time of the study, Australia had a population of 20 million people and recorded 206,000 live births.

The most detailed source of data on the costs of folic acid fortification for a developing country comes from South Africa, which mandated fortification in 2003 of maize and wheat flour. Health South Africa report that a grant from GAIN of $2,800,000 was used to cover the following upfront costs over a 3‐year period (2004–2007): $1,050,000 for production equipment; $750,000 for social marketing and communications; $650,000 for monitoring and evaluation; and $350,000 for program management and implementation. The cost for production equipment largely went to subsidize the purchase of fortification equipment on a sliding scale, with 100% reimbursement for small millers, 75% for medium‐sized millers, and 50% for large millers.^13^ It is not clear whether these costs applied only to folic acid fortification or to a wider set of fortification efforts that included iron, thiamine, and zinc. It is also not clear whether there were subsequent expenditures on social marketing or compliance. Sayed *et al*. estimate that the annual cost of purchasing folic acid for inclusion in premix, with fortification levels set at 1.5 mg folic acid per 1.5 kg wheat flour and 2.1 mg folic acid per 2.21 kg maize flour, was approximately $200,000 per year for a country of, in 2006, 46 million people.^9^

The cost of folic acid itself depends on three factors: (1) population, (2) the level of consumption of fortified foods, and (3) the price of folic acid. Berry *et al*. show that going from low levels of flour intake (<75 g per person per day) to high (150–300 g) reduces cost by ∼70% per metric ton.^14^ While it appears that the prediction made by Berry *et al*. that folic acid prices would rise over time has not been borne out following the decision of a number of Chinese firms to begin producing folic acid, there have been considerable fluctuations in price. Grosse *et al*. report that the unit cost of adding folic acid to the premix used by U.S. millers was $0.10–0.15 per metric ton of flour in 2013 and $0.20–0.30 per metric ton in 2014 before rising to $1.00 per ton in May 2015 following the temporary cessation of production by several Chinese firms.^10^, ^15^ By late 2015, prices had fallen to approximately $0.50 per ton.^15^ The important point to take from this discussion of costs is that folic acid fortification is not expensive; in fact, it is cheap. To put these figures in context, at the peak of folic acid prices in 2015, the recurrent cost of folic acid fortification in the United States, with its population of approximately 320 million people, was estimated to be $20 million or $0.065 per person.^10^ By contrast, total bilateral aid for health and education in 2015 to developing countries was approximately $25 billion.^16^

### Comparing benefits and costs

There are a number of ways in which these estimates of benefits and costs can be brought together, including the cost of averted mortality, the cost of averting a disability‐adjusted life year (DALY), and benefit:cost ratios (BCRs).

Data presented by Grosse *et al*. show that BCRs for folic acid fortification range from 22 to 110 in the United States.^10^ In South Africa, Sayed *et al*. estimate a BCR of 30.^9^ For Australia, estimates vary by the level of fortification and assumptions regarding the monetary value of averted NTDs; these range from 4.5 to 37.8.^12^ Across these three studies, BCRs always exceed 1, indicating that they are a good investment. But the wide range in their values—for these studies there is a 25‐fold difference between the lowest and highest estimate—suggests that these are not unproblematic. One problem in comparing the three studies listed above is that they include different benefits and costs in their calculations; they are not, appearances notwithstanding, directly comparable. A second problem, particularly relevant to NTDs, is that they are sensitive to the availability, quality, and cost of care of NTD cases. Consider two developing countries: one (country A) where there is some medical care for spina bifida and another (country B) where no care exists. Holding all other benefits and costs constant, the BCR for folic acid fortification for country A will be higher than for country B because the benefit for country A will include the averted healthcare costs. This produces some uncomfortable logic, namely that, given limited resources for fortification, priority should be given to countries where there is medical care for NTDs; countries where such care is lacking should receive lower priority. Such logic effectively penalizes poorer countries, and it is not obvious in the case of folic acid fortification that doing so is appropriate. While in principle it is possible to value averted mortality in terms of the monetary value of a life saved, setting these monetary values is fraught with ethical and practical problems. Given all this, it makes more sense to compare the benefits and costs of folic acid fortification using the cost of averting deaths or the costs of averted DALYs. To do so, we need to specify a time frame over which these benefits and costs are calculated, given that the costs are both upfront and recurring. We assume a 10‐year time horizon.

The cost of averting a death requires data on the reduction in stillbirths and infant and child mortality resulting from fortification with folic acid. For our best estimate, we take the most conservative published estimate from South Africa, which, as discussed above, saw a reduction of 0.43 NTDs/1000 births following the introduction of folic acid fortification,^9^ make no allowance for averted stillbirths, and round to a reduction of 0.40 NTDs/1000 births. We can construct more optimistic estimates using values that are multiples of our best estimate, such as 1.5 or 2, or more pessimistic estimates by using multiples such as 0.5.

Cost calculations require assumptions about what costs will be incurred, by whom, and over what period. There are little developing country data on this; one exception being South Africa. The upfront public sector costs in South Africa were $2,800,000 over the 3‐year period 2004–2006. During this period, there were approximately 4.2 million live births in South Africa.^17^ This expenditure is equivalent to $0.66 per live birth in 2004–2006; accounting for inflation, this equals $0.80 in 2017 U.S. dollars. Assume that, for the first 3 years of folic acid fortification, comparable costs (which cover social marketing, subsidies to millers to buy equipment, and administrative costs associated with quality testing and establishing monitoring and compliance systems) are incurred, but, unlike in South Africa, recurrent costs are budgeted at $0.20 per live birth for the following 7 years to cover ongoing social marketing, monitoring and compliance costs, and possibly a subsidy for the importation of folic acid. Over a 10‐year period, this comes to $3,830,000 given 1,000,000 births per year. Note that these are all costs are assumed to be borne by the public sector.

We can also consider two alternative cost scenarios. In the high‐cost case, expenses for the first 3 years are higher, at $1.00 per live birth. They fall gradually to $0.60 per live birth for years 4–6 and then to $0.30 for years 7–10. This scenario can be considered applicable to a country where there are limited existing fortification efforts (so the upfront costs are higher) and where there is a need for more intensive monitoring of implementation. In the low‐cost case, expenses for the first 3 years are lower at $0.60 per live birth before falling to $0.15 per live birth for years 4–10. This scenario would be applicable to a country where, because some foods are already fortified, subsidies to millers and the costs associated with modifying the testing, monitoring, and compliance systems would be less.

For DALYs, we first need to estimate the DALYs saved as a result of fortification. This requires an accurate assessment of the existing prevalence of NTDs, what fraction of the target population would consume the fortified products, and what quantities of these products they would consume (taking into account the fact that, if fortification leads to higher prices, some of the target population might shift consumption to nonfortified substitutes). For many countries that do not fortify with folic acid, limited information is available on these parameters; furthermore, it is not obvious that information from fortification experiences in developed countries (with their different patterns of staple food consumption) is relevant for poorer, less urbanized developing countries. Again, the exception to this is South Africa, which, as discussed above, saw a reduction of 0.43 NTDs/1000 births following the introduction of folic acid fortification.^9^

Assuming that a birth with an NTD always leads to rapid death, for the DALY calculation, we also need to know the number of live births and the life expectancy. As an illustration, consider Zambia, a country in southern Africa that does not mandate folic acid fortification. The main staple is maize, which is consumed in the form of porridge prepared from maize flour; maize flour is fortifiable. Approximately 40% of the population lives in urban areas. Life expectancy is 60 years, and there are 670,000 live births per year. Assuming that the experience of South Africa would carry over to Zambia, folic acid fortification of maize flour in Zambia would avert 17,286 DALYs per year (670,000 live births × an NTD reduction of 0.43 NTDs/1000 live births × 60) or 172,860 DALYs over a 10‐year period.

As we did with the cost of averting a death, cost calculations require assumptions about what costs will be incurred, by whom, and over what period. Again, using the South African costs as a basis, these were $0.80 in 2017 U.S. dollars. We can assume that, for the first 3 years of folic acid fortification in Zambia, comparable costs were incurred, but, unlike in South Africa, recurrent costs are budgeted at $0.20 per live birth for the following 7 years to cover ongoing social marketing, monitoring, and compliance costs and a subsidy for the importation of folic acid. Over a 10‐year period, these costs come to $2,576,000 ($0.80 per live birth × 670,000 live births × 3 years + $0.20 per live birth × 670,000 live births × 7 years).

## Results

Table 1 presents a comparison of these costs and deaths averted over a 10‐year period (to make the numbers easier to work with, it assumes 1,000,000 births per year) and a range of estimates using the base cost case and different estimates of deaths averted per 1000 live births. Our best estimate is that, with mandatory fortification, the cost per death averted is $957. This estimate is lower when we are more optimistic about the effect of fortification on averting deaths; for example, it is only $319 when we assume that 1.2 deaths are averted per 1000 live births. It is higher ($1915) when we are more pessimistic about the number of deaths averted.

**Table 1:** Estimates of cost per death averted (10‐year period, 1,000,000 live births, base cost case) by scenario

Scenario	Deaths averted (per 1000 live births)	Number of deaths averted	Cost (USD)	Cost (USD) per death averted
(1) Most pessimistic	0.2	2000	$3,830,000	$1915
(2) Best estimate	0.4	4000	$3,830,000	$957
(3)	0.6	6000	$3,830,000	$638
(4)	0.8	8000	$3,830,000	$478
(5)	1.0	10,000	$3,830,000	$383
(6) Most optimistic	1.2	12,000	$3,830,000	$319

SOURCE: Author calculations.

Table 2 shows the estimates of the cost per death averted under the six scenarios (most pessimistic to most optimistic) used in Table 1 as well as alternative assumptions about costs (high cost and low cost). These alternative assumptions extend the range of values associated with mandatory folic acid fortification. The lowest estimate ($240) comes from the most optimistic scenario regarding the impact of fortification, a reduction of 1.2 deaths per 1000 live births, together with the low‐cost scenario. The highest estimate ($3015) comes from the most pessimistic scenario regarding the impact of fortification, a reduction of 0.2 deaths per 1000 live births, together with the high‐cost scenario.

**Table 2:** Estimates of cost per death averted (10‐year period, 1,000,000 live births) by scenario and cost assumptions

	Cost (USD) per death averted		
Scenario	High cost	Base cost case (from Table 1)	Low cost
(1) Most pessimistic	$3015	$1915	$1440
(2) Best estimate	$1508	$957	$720
(3)	$1005	$638	$480
(4)	$754	$478	$360
(5)	$603	$383	$288
(6) Most optimistic	$503	$319	$240

SOURCE: Author calculations.

To put these numbers into perspective, Rheingans *et al*. estimate that a lower‐bound cost per death averted for the rotavirus vaccine is $3015.^18^ Using the LiST model, Eisele *et al*. estimated that the scale‐up of insecticide‐treated bed nets and other malaria‐prevention activities in sub‐Saharan Africa between 2000 and 2010 cost $2770 per death averted.^19^ Even under our most conservative assumptions regarding costs and impact, mandatory folic acid fortification compares favorably to these other widely adopted life‐saving interventions.

For Zambia, applying the methods described above gives an averted DALY per dollar spent on folic acid fortification of $14.90. This is a conservative figure in the sense that it assumes a relatively modest reduction in NTDs following the introduction of folic acid fortification; the South African figures used here are lower than in the other countries reported by Castillo‐Lancelotti *et al*.^8^ and also lower than recent results reported from a folic acid fortification randomized controlled trial in a poor region of China.^20^ They are conservative in that they assume that new equipment would be needed by millers, social marketing would be required, and that these stand‐alone costs not shared with simultaneous efforts to fortify other foods. They are also conservative in that they use Zambia‐specific and not standardized life expectancy. They are liberal in the sense that they assume that all cases of NTDs lead to immediate death; if some survivorship is assumed, the averted DALYs would be lower.^20^ Given the relatively short time horizon over which these averted DALYs and costs are calculated, neither are discounted.

Is $14.90 a high or low cost? For Zambia, it is possible to compare it against averted DALYs per dollar spent on other forms of food fortification and, more generally, against other life‐saving interventions relevant to a poor, southern African country. Table 3 shows that, based on the assumptions described above, folic acid fortification of maize flour has a lower cost per DALY averted than nearly all other forms of fortification and is lower than two other widespread interventions—insecticide‐treated bed nets and treatment for severe acute malnutrition—aimed at averted infant and child mortality. Table 4 shows the sensitivity of this calculation to changes in the impact on NTD‐related deaths and to changes in the costs of introducing mandatory folic acid fortification. Again, a 10‐year time frame is used for a country like Zambia with 670,000 live births per year. The cost per averted DALY from mandatory folic acid fortification is low compared with the alternatives described in Table 3.

**Table 3:** Comparing hypothetical folic acid fortification in Zambia against alternative investments as averted DALYs per dollar spent

Study	Intervention	Cost per DALY averted (USD)
	Hypothetical folic acid fortification in Zambia	$14.90
Alternative fortification investments		
Fiedler and Lividini^21^	Food fortification, vitamin A, vegetable oil: Zambia	$8.00
Fiedler and Lividini^21^	Food fortification, vitamin A, sugar: Zambia	$20.00
Fiedler and Lividini^21^	Food fortification, vitamin A, wheat flour: Zambia	$40.00
Horton^22^	Food fortification, iron: East Africa	$25.00
Horton^22^	Food fortification, vitamin A: East Africa	$35.00
Horton^22^	Food fortification, zinc: East Africa	$60.00
Alternative health‐related investments		
Pulkki‐Brannstrom *et al*.^23^	Long‐life insecticide‐treated bed nets	$16.80
Shekar *et al*.^24^	Treatment for severe acute malnutrition (SAM) (calculated from table 6.2 in Ref. 24, assuming life expectancy of 60)	$175.00

**Table 4:** Comparing averted DALYs per dollar spent: sensitivity analysis

Scenario	Intervention	Cost per DALY averted (USD)
Base case	Hypothetical folic acid fortification in Zambia	$14.90
Relative to base case		
High effectiveness (increase impact by 50%)	Costs: $0.80/birth for years 1–3 $0.20/birth for years 4–10 Impact: ↓ NTD by 0.645/1000 births	$9.93
Low effectiveness (reduce impact by 50%)	Costs: $0.80/birth for years 1–3 $0.20/birth for years 4–10 Impact: ↓ NTD by 0.215/1000 births	$29.80
High cost (increase cost per year in years 1–3 by 25%; more gradual cost reductions in years 4–10)	Costs: $1.00/birth for years 1–3 $0.60/birth for years 4–6 $0.30/birth for years 7–10 Impact: ↓ NTD by 0.430/1000 births	$23.43
Low cost (reduce cost per year by 25%)	Costs: $0.60/birth for years 1–3 $0.15/birth for years 4–10 Impact: ↓ NTD by 0.430/1000 births	$11.22
High cost (increase cost per year in years 1–3 by 25%; more gradual cost reductions in years 4–10) Low effectiveness (reduce impact by 50%)	Costs: $1.00/birth for years 1–3 $0.60/birth for years 4–6 $0.30/birth for years 7–10 Impact: ↓ NTD by 0.215/1000 births	$46.86

SOURCE: Author calculations.

## Discussion

We constructed an investment case comparing the costs and benefits of folic acid fortification relative to alternative life‐saving investments and using these to inform estimates of the financing required for implementation. Our best estimate is that the cost per death averted through mandatory folic acid fortification is $957 and the cost per DALY is $14.90. Both compare favorably to recommended life‐saving interventions, such as the rotavirus vaccine and insecticide‐treated bed nets. Thus, there is a strong economic argument for mandatory folic acid fortification.

There are three caveats to this investment case. These relate to price changes, adherence to regulatory standards, and supplementation as an alternative to fortification.

In the long run, increases in the cost of production are passed on to consumers in the form of higher prices; higher prices lead to reduced consumption. The magnitude of this reduced consumption depends on the magnitude of the price change, the price sensitivity of consumers, and the availability of substitutes. Reduced consumption of fortified products will attenuate the impact of folic acid fortification.

In principle, this should not be a concern. As discussed above, folic acid is not expensive, and there are ways in which its cost can be reduced—for example, through bulk purchases of folic acid by millers associations that are then distributed to individual firms, through exempting imports of folic acid from import taxes, exempting locally fortified foods from value‐added tax for sales taxes, or through the inclusion of folic acid in premix used for iron, zinc, or other flour fortificants. The Food Fortification Initiative estimates that fortification increases the price of 1 kg of flour by $0.0006 or 0.16% of current retail price; for 1 kg of fortified rice, it ranges from $0.08 to $0.16 per 10 kg of rice or 1.5–3% of retail prices^25^ (it is not clear whether these figures refer solely to folic acid fortification or to fortification more generally). The Food Safety and Standards Authority of India (FSSAI) reports that the cost for fortification of 1 kg of wheat flour with iron, folic acid, and vitamin B_12_ is approximately 3–15 paisa.^26^ In the latter half of 2016, retail wheat flour prices in India ranged from R22 to R26, yielding an upper bound estimate of fortifying wheat flour (15 paisa/R26) as 0.6%. However, mandatory fortification creates a barrier to entry for new food processors, as they need to purchase the equipment needed for fortification. This gives (larger) market incumbents an advantage—they are protected from domestic competition and from nonfortified imports, and this may also lead to higher prices. In fact, case studies of fortification of other products suggest that this is one reason why large food‐processing firms in developing countries find fortification attractive.^27^ These higher prices may cause consumers, especially poorer consumers, to continue consuming or shift to nonfortified foods, potentially attenuating the impact of fortification. For example, if maize prices were to rise by 10% in southern Africa following fortification, demand for maize would fall by 5%, and demand for (nonfortifiable) cassava would rise by 3%.

It is not clear how significant the attenuating effects of price increases will be in practice. Should mandatory fortification create barriers to entry, one policy response would be to provide new entrants with the technical assistance (and possibly subsidies) needed to ensure that they can fortify their products.

Passing legislation requiring mandatory fortification does not mean that fortification takes place. Aaron *et al*. assessed 18 large‐scale food fortification programs in eight countries, finding that only two met program criteria for coverage.^28^ Luthringer *et al*. report that, across the 20 programs and 12 countries considered in their study, quality assurance data show that less than half the samples are adequately fortified against national standards.^29^ They note multiple reasons why adherence to regulatory fortification standards was low, including poor capacity to enforce existing regulations (because the regulations themselves are unclear and limited human resources, limited funding, and inadequate laboratory capacity), limited penalties for compliance (low probabilities of being caught as noncompliant, low fines), and governance and political economy considerations. In environments where consumers are unable to detect quality differences, and regulatory systems are weak, it is not difficult for unscrupulous firms to undercut those firms following the rules by offering their products at a lower price while falsely claiming that they have been fortified. In the case of folic acid, nonadherence reduces the effectiveness of fortification, which in turn lowers the number of averted deaths and DALYs; this is the justification for recurrent funding to improve monitoring and encourage compliance. However, it also opens the question of whether, at the margin, it is more cost‐effective to fund expansion of mandatory folic acid fortification to new countries or to fund adherence efforts in those countries where legislation is already in place and where there is a political willingness (but not funding or technical capacity) to enforce fortification.

Successful fortification requires that women consume the fortified product in sufficient quantities that their intake of folic acid is sufficient to prevent NTDs. In developing countries, especially in rural areas where food markets may be absent or inaccessible, women, men and children may not consume foods that have been processed, instead consuming their own production of staple and other foods,^30^ or they may consume products from small millers who may not be covered by mandatory fortification. Such consumers are unlikely to be reached by folic acid fortification of flours. Is supplementation an economically viable alternative? On practical grounds, this seems unlikely. For folic acid supplementation to be effective against NTDs, it would need to be taken continuously for the full period of reproductive age. Even in a country like Bangladesh, which is well advanced in terms of its demographic transition and where relatively few women give birth after age 30, this would be continuous supplementation for 12 years. Furthermore, women not reached by fortified foods are more likely to live in more remote areas where it may be challenging to ensure a continuous supply of folic acid supplements. For this reason, we do not attempt to construct an investment case for folic acid supplementation (on this, also see Ref. 24).

There is a strong economic argument for mandatory folic acid fortification. It is likely that folic acid fortification will yield a positive return on investment for societies and prevent thousands of preventable child deaths. More precise estimates require better data on the costs of implementing fortification and on the costs of improving compliance where regulations are already in place.

## Competing interests

The author declares no competing interests.
